# Psychometric evaluation of the Functional Health Pattern Assessment Screening Tool – Modified Brazilian Version

**DOI:** 10.1590/1518-8345.6755.4119

**Published:** 2024-03-15

**Authors:** Rita de Cassia Gengo e Silva Butcher, Lidia Santiago Guandalini, Alba Lucia Bottura Leite de Barros, Bruna Bronhara Damiani, Dorothy Anne Jones

**Affiliations:** ^1^ Florida Atlantic University, Christine E. Lynn College of Nursing, Boca Raton, FL, United States of America.; ^2^ Sanford Health Center, Fargo, ND, United States of America.; ^3^ Universidade Federal de São Paulo, Escola Paulista de Enfermagem, São Paulo, SP, Brazil.; ^4^ Estatikos Consultoria Estatística, São Paulo, SP, Brazil.; ^5^ Boston College, William F Connell School of Nursing, Chesnut Hill, MA, United States of America.

**Keywords:** Nursing Assessment, Clinical Reasoning, Nursing Process, Validation Study, Psychometrics, Nursing, Evaluación en Enfermería, Razonamiento Clínico, Proceso de Enfermería, Estudio de Validación, Psicometría, Enfermería, Avaliação em Enfermagem, Raciocínio Clínico, Processo de Enfermagem, Estudo de Validação, Psicometria, Enfermagem

## Abstract

**Objective:**

to test the factorial structure, reliability and convergent validity of the Functional Health Pattern Assessment Screening Tool – Modified Brazilian Version.

**Method:**

this was a psychometric evaluation of the Functional Health Pattern Assessment Screening Tool – Modified Brazilian Version. Seven hundred and seventeen participants answered the data collection instrument consisting of two parts. Part I included a structured questionnaire to collect sociodemographic data and the participants’ perceptions and satisfaction with their current health status. Part II consisted of the tool being tested. The internal structure was assessed using Confirmatory Factor Analysis. Convergent validity was evaluated by the correlation of the tool scores with the rates corresponding to self-perception and satisfaction with current health status. Reliability was assessed using Cronbach’s alpha.

**Results:**

the Confirmatory Factor Analysis confirmed a three-factor solution. The factor loadings were significant and varied from 0.16 to 0.75; the fit indices suggested moderate fit of the model. Internal consistency for all three components varied between 0.779 and 0.919.

**Conclusion:**

the findings suggest that the tool is valid and reliable to be used in the Brazilian population, although caution is recommended when interpreting the results due to the moderate fit of the model.

## Resumen

**Objetivo:**

someter a prueba la estructura factorial, confiabilidad y validez convergente del instrumento *Functional Health Pattern Assessment Screening Tool* – Versión modificada para Brasil.

**Método:**

evaluación psicométrica del instrumento *Functional Health Pattern Assessment Screening Tool* – Versión modificada para Brasil. Setecientos diecisiete participantes respondieron el instrumento de recolección de datos, compuesto por dos partes. La Parte I incluyó un cuestionario estructurado para recopilar datos sociodemográficos y las percepciones y el nivel de satisfacción de los participantes con respecto a su estado de salud actual. La Parte II consistió en la herramienta sometida a prueba. La estructura interna se evaluó empleando Análisis Factorial Confirmatorio. La validez interna se evaluó por medio de la correlación entre las puntuaciones obtenidas en la herramienta y los índices correspondientes a los niveles de autopercepción y satisfacción con respecto al estado de salud actual. La confiabilidad se evaluó utilizando el coeficiente alfa de Cronbach.

**Resultados:**

el Análisis Factorial Confirmatorio confirmó una solución con tres factores. Las cargas factoriales fueron significativas y variaron entre 0,16 y 0.75; los índices de ajuste sugirieron ajuste moderado del modelo. La consistencia interna correspondiente a los tres componentes varió entre 0,779 y 0,919.

**Conclusión:**

los hallazgos sugieren que la herramienta es válida y confiable para ser usada en la población de Brasil, aunque se recomienda interpretar los resultados con precaución debido al moderado ajuste del modelo.

## Resumo

**Objetivo:**

testar a estrutura fatorial, a confiabilidade e a validade convergente do *Functional Health Pattern Assessment Screening Tool* – Versão Brasileira Modificada.

**Método:**

avaliação psicométrica do *Functional Health Pattern Assessment Screening Tool* – Versão Brasileira Modificada. Setecentos e dezessete participantes responderam os itens do instrumento de coleta de dados composto por duas partes. A Parte I incluiu um questionário estruturado contendo dados sociodemográficos e a percepção e satisfação dos participantes com seu estado de saúde atual. A Parte II consistiu no instrumento testado. A estrutura interna foi avaliada por meio de Análise Fatorial Confirmatória. A validade convergente foi avaliada pela correlação dos escores do instrumento com os índices correspondentes à autopercepção e à satisfação com o estado de saúde atual. A confiabilidade foi avaliada pelo alfa de *Cronbach*.

**Resultados:**

a Análise Fatorial Confirmatória confirmou uma solução de três fatores. As cargas fatoriais foram significativas e variaram de 0,16 a 0,75; os índices de ajuste sugeriram ajuste moderado do modelo. A consistência interna dos três componentes variou entre 0,779 e 0,919.

**Conclusão:**

os achados sugerem que o instrumento é válido e confiável para ser utilizado na população brasileira, embora seja recomendada cautela na interpretação dos resultados devido ao ajuste moderado do modelo.

**Highlights:**

**(1)** The FHPAST-BR is a structured, valid and reliable Nursing-driven assessment tool.

**(2)** The tool provides a way of organizing clinical data and easing decision-making.

**(3)** The FHPAST-BR can be used in clinical practice and research.

## Introduction

Nursing assessment is paramount to nursing practice, as it informs clinical decision-making and patient-centered care ^(^ ^1^ ^-^ ^2^ ^)^. Structured Nursing assessments are recognized as important tools for providing high-quality, safe and cost-effective Nursing care ^(^ ^3^ ^)^. Several structured Nursing assessment tools are available ^(^ ^4^ ^-^ ^6^ ^)^. However, the specific knowledge and focus of the Nursing discipline are not always present in Nursing assessment tools ^(^ ^6^ ^)^. Lacking discipline-specific models and frameworks to organize data collection imposes a challenge for nurses to express the nature and goals of Nursing and make the discipline visible ^(^ ^7^ ^-^ ^8^ ^)^. The Functional Health Pattern Assessment Screening Tool (FHPAST) is a Nursing assessment screening instrument grounded in the Functional Health Patterns (FHP) framework ^(^ ^9^ ^)^. There is evidence showing the sound psychometric properties of the FHPAST in the United States and Spain ^(^ ^8^ ^-^ ^12^ ^)^. However, the tool has not been validated for use in Brazilian Portuguese. 

In this study, Nursing assessment is defined as a comprehensive, dynamic and systematic process that takes place during a deliberate encounter between the nurse and the patient, whereby clinical information is synthesized as human responses ^(^ ^9^ ^)^. A comprehensive Nursing assessment provides nurses with the opportunity to come to know the patients as whole human beings, as well as to identify nurse-focused phenomena of concern, leading to the selection and implementation of appropriate interventions ^(^ ^11^ ^)^. The association between Nursing assessment data and both patient- and setting-related outcomes has been shown in the literature ^(^ ^12^ ^)^. Moreover, Nursing assessment has been linked to higher patient satisfaction with services provided ^(^ ^13^ ^)^. 

The FHP framework provides a structure to guide nursing assessment, clinical decision-making and Nursing care. The assessment process sheds light on the substantive Nursing content, clinical practice and disciplinary knowledge ^(^ ^9^ ^)^. The framework was developed in the early 1980s and has been used globally by nurses in the clinical practice, education and research ^(^ ^6^ ^,^ ^8^ ^,^ ^14^ ^-^ ^17^ ^)^. Eleven FHPs have been described: Health Perception-Health Maintenance, Nutrition-Metabolic, Elimination, Activity-Exercise, Sleep-Rest, Coping-Stress, Self-Concept-Self-Perception, Cognitive-Perceptual, Role-Relationship, Sexuality-Reproductive, and Value-Belief ^(^ ^9^ ^)^. 

Despite its relevance, some nurses consider using the FHP framework to guide nursing assessment as time consuming, especially in outpatient and community health environments. Considering that time constraints are a barrier to using such framework, the FHPAST was developed to provide a reliable and valid self-report tool to screen functional health patterns across populations and settings ^(^ ^8^ ^)^. This instrument is a self-report screening tool that can be independently completed by the person being assessed. The screening items were generated using the definition of each FHP ^(^ ^9^ ^)^ and related literature. Originally, the tool consisted of 83 items written in declarative statements answered using a 4-point Likert scale (“never” to “routinely”) to reflect the patient’s perceived behavior over the past four weeks. 

The tool was initially tested in Nursing students, resulting in the removal of 25 items. Further psychometric testing of the 58-item tool using Principal Component Analysis led to removing one item and revealed a three-factor solution. Reliability of the overall 57-item tool was satisfactory (Cronbach’s alpha = 0.92), as well as for each factor (Cronbach’s alpha from 0.78 to 0.97) ^(^ ^8^ ^)^. 

The 58-item version of the FHPAST was translated into Brazilian Portuguese. Reliability of the translated version was satisfactory (Cronbach’s alpha = 0.94). However, participants needed help to understand the items and complete the assessment using the FHPAST. Thus, the authors recommended language revisions to allow for a more culturally sensitive tool. Furthermore, the FHPAST lacks robust evidence of validity in the Brazilian population ^(^ ^18^ ^)^. 

The objective of this psychometric study was to test the factorial structure, reliability and convergent validity of the Functional Health Pattern Assessment Screening Tool – Modified Brazilian Version.

## Method

### Design

This study was developed following the best practices for developing and validating scales in health, social and behavioral research ^(^ ^19^ ^)^. Permission was obtained by email from the original authors to use the FHPAST in developing the Functional Health Pattern Assessment Screening Tool – Modified Brazilian Version (FHPAST-BR). 

Prior to the psychometric validation, a revision of the previous Portuguese translation of the FHPAST and a content validation process were performed. The Brazilian authors of the translated 58-item FHPAST into Portuguese (T1) were contacted and, during an online meeting, granted permission to revise the initial T1 translation. Noteworthy, such translation followed criteria duly established in the literature ^(^ ^20^ ^)^. 

In this study, the principal investigator revised the translation of 57 items that comprise the latest English version of the FHPAST. This revision resulted in content updates of two items to reflect the most recent recommendations regarding physical activity and consumption of a healthy diet ^(^ ^21^ ^)^. Thus, the item “I do aerobic exercise for 20 min 2-3 or more times a week” was initially reworded to “I do at least 2.5 hours of physical activity every week”, and the item “I intentionally limit my dietary fat intake” to “I control the types of food I eat”. 

The remaining 55 items underwent a lexical revision. Its purpose was to identify and use words and expressions with good representation in the lexicon of the Brazilian population with varying levels of education and health literacy. According to the Lexical Quality Hypothesis, the quality of a word’s representation is determined by the reader’s knowledge of that word, which is influenced by frequency, family size and spelling ^(^ ^22^ ^)^. Thus, except for pronouns, prepositions, conjunctions and articles, all other words from each item were analyzed in terms of structure, extension, complexity and frequency. Word frequency was consulted in the free online version of *Corpus Brasileiro* ^(^ ^23^ ^)^. The lexical revision resulted in rewording of 25 items (1, 10, 14, 15, 20-23, 28-31, 35, 38, 39, 41, 43-45, 47, 48, 52, 53, 55 and 56). 

All 57 items in FHPAST-BR were analyzed by four experts and ten individuals from the target population. The number of experts and individuals from the target population was determined based on the literature ^(^ ^24^ ^-^ ^26^ ^)^. The items that maintained the original translation (T1) and the updated ones were analyzed by experts according to “relevance” (the item assesses one FHP), and the reworded items were analyzed according to “relevance” and “precision” (the item has the same meaning as in T1) ^(^ ^27^ ^)^. All items were validated by the experts. Subsequently, patients analyzed each item for comprehensibility. All items were understood by at least 70% of the patients. This criterion was based on the researchers’ previous experience (data not published). Similar cutoff points for comprehensibility have been used in recent publications ^(^ ^28^ ^)^. Suggestions made by the patients to improve clarity and comprehensibility were incorporated into the items. This version was submitted to Confirmatory Factor Analysis. 

### Study setting

This study was conducted at a public university hospital in São Paulo, SP, Brazil.

### Period

The data were collected between February and December 2020.

### Population

The population consisted of patients, significant others, faculty, staff and students present at the hospital during the data collection period. We defined the study population composition to resemble that of the FHPAST validation in the United States, which consisted of patients from “an outpatient rheumatology practice, an ophthalmology clinic, and three health fairs on a private college campus” ^(^ ^8^ ^)^. 

### Selection criteria

All individuals 18 years or older were included in the study. Eligible individuals were invited to participate in the study by a trained research assistant who obtained written consent from those that agreed to participate in the study and provided them with a printed data collection form.

### Sample

The sample was defined as at least 10 participants per item in the FHPAST-BR ^(^ ^29^ ^)^. Thus, it was calculated at a minimum of 570 participants. The sample consisted of 717 participants. 

### Study variables

Sociodemographic variables (age, race/ethnicity, gender, education, employment) were chosen to characterize the sample. To assess participants’ perception and satisfaction with their current health status and to analyze convergent validity, the FHPAST-BR was used.

### Instrument used to collect information

The data collection instrument had two parts. Part I consisted of a structured questionnaire to collect sociodemographic data and participants’ perceptions and satisfaction with their current health status. Self-perception and satisfaction were assessed through the following questions: “Overall, how would you rate your current health?” and “How satisfied are you with your current health?”. A 6-point scale followed each question, varying from Excellent to Poor and from Completely satisfied to Completely unsatisfied. Part II consisted of the FHPAST-BR itself.

Each item in the FHPAST-BR is a declarative sentence followed by a Likert scale which was changed from a 4-point scale ^(^ ^8^ ^)^ to a 5-point one in FHPAST-BR, including the Never (1), Almost never (2), Sometimes (3), Almost always (4), and Always (5) options. The 5-point scale added a midpoint to the continuum from Never to Always and gave participants a fair number of options to rate their opinion ^(^ ^29^ ^)^. Participants should answer each item based on their behaviors within the past four weeks. The change in the Likert scale was made for the first time in this study, and the authors of the original FHPAST agreed with it. Reverse coding of items 43–58 is required ^(^ ^8^ ^)^. The final score is given by the mean value among all items. In the FHPAST-BR, a mean score greater than or equal to 4 suggests functional health. In order to compare the results from the FHPAST-BR to the English version, the final FHPAST-BR score should be multiplied by 0.80. 

### Data collection

All data were collected in-person. A research assistant explained the objectives and data collection procedures to all potential participants. Those who agreed to participate were asked to sign a written consent form. Subsequently, the participants were asked to complete the two-part study instrument. Therefore, the FHPAST-BR data were obtained by self-report.

### Data treatment and analysis

The analysis was performed using the R software. Descriptive statistics were used to present the characteristics of the sample. Data quality was evaluated by means of descriptive statistics in terms of the score distribution of the items and missing data. Missing observations 218 were dismissed, and only 499 participants who answered all the items remained in the analysis.

In the Confirmatory Factor Analysis (CFA), the three-factor structure was tested, the same structure tested for the English version of the FHPAST. The three factors are the following: Health Risk/Threat (items 41 to 57), General Well-Being & Self-Confidence (items 1, 3-6, 8, 12-19, 21, 26-31, 34-38 and 40), and Health Promotion/Protection Activity (items 2, 7, 9, 10-11, 20, 22-25, 32-33 and 39). The CFA was adjusted using the items’ covariance matrix. Factor loadings higher than 0.30 were considered adequate ^(^ ^8^ ^,^ ^30^ ^)^. 

The CFA fit model was evaluated with absolute, parsimonious and incremental indices. The Standardized Root Mean Square Residual (SRMR) is an absolute index in which the lower the value, the better the fit of the model. The Root Mean Square Error of Approximation (RMSEA) is a parsimonious fit index, where values ≤0.05 indicate good fit of the model. The Confirmatory Factor Index (CFI) and the Tucker Lewis Index (TLI) are incremental fit indices, and values >0.90 indicate good fit for the model of interest. In addition, the correlation coefficient between domains was analyzed.

Cronbach’s alpha was calculated to evaluate internal consistency reliability in each domain. Alpha values above 0.70 indicate satisfactory internal consistency ^(^ ^31^ ^)^. Finally, Spearman’s correlation was used for convergent validity to test both hypotheses: 1) The higher the FHPAST-BR score, the better the current health perception; and 2) The higher the FHPAST-BR score, the better the satisfaction with current health. It was expected that the scores would be at least moderately (r > 0.40) and positively correlated. 

### Ethical considerations

The Ethics Committee approved the study. All research procedures were guided by national and international regulations for research involving human beings. All participants signed a consent form. Confidentiality was assured, as no identification data were collected from the participants, and the consent forms were stored separately from the data collection instruments.

## Results

### Characteristics of the sample

The characteristics of the sample are described in Table 1. In total, 499 participants answered all items and were included in the analysis. 

**Table 1 -:** Sociodemographic characteristics and participants’ perception and satisfaction with their current health status (n = 499). São Paulo, SP, Brazil, 2020

**Characteristics**	
Age (years old), mean (SD*)	34,1 (10,8)
Gender, n ^†^ (%)	
Female	366 (73,3%)
Male	133 (26,7%)
Self-reported skin color, n ^†^ (%)	
White	264 (52,9%)
Non-white	232 (46,5%)
Not reported	3 (0,6%)
Education, n ^†^ (%)	
Lower than Elementary School	19 (3,8%)
Elementary School	42 (8,4%)
High School	279 (55,9%)
University Degree	159 (31,9%)
Employed, n ^†^ **(%)**	
No	144 (28,9%)
Yes	355 (71,1%)
Type of participant, n ^†^ **(%)**	
Patients	246 (49,3%)
Significant others	217 (43,5%)
Faculty/Staff/Students	35 (7,0%)
Not reported	1 (0,2%)
Current health status perception ^‡^ , n ^†^ **(%)**	
Excellent	41 (8,2%)
Very good	80 (16,0%)
Good	79 (15,8%)
Fairly good	203 (40,7%)
Fair	87 (17,4%)
Poor	8 (1,6%)
Not reported	1 (0,2%)
Current health status satisfaction ^§^ , n ^†^ **(%)**	
Completely unsatisfied	12 (2,4%)
Very unsatisfied	32 (6,4%)
Somewhat unsatisfied	86 (17,2%)
Completely satisfied	47 (9,4%)
Very satisfied	179 (35,9%)
Somewhat satisfied	142 (28,5%)
Not reported	1 (0,2%)

^*^ SD = Standard Deviation;

^†^ n = Sample Size;

^‡^ n = 715;

^§^ n = 714

### Data quality and homogeneity

The level of missing data was low, between 1 (0.1%) and 10 (1.7%), except for items 34 (“I am satisfied with what I do for work”) and 44 (“I feel guilty when I drink alcohol, wine, or beer”), whose levels of missing data were 64 (8.9%) and 86 (12.0%). All answer categories were used. The number of answers for the Never (1) category varied between 1 and 435; for Almost never (2), between 4 and 130; for Sometimes (3), between 18 and 179; for Almost always (4), between 9 and 190; and for Always (5), between 6 and 401. Table 2 shows the item statistics for the FHPAST-BR. 

**Table 2 -:** Items’ distribution for the Functional Health Pattern Assessment Screening Tool – Modified Brazilian Version (n = 499). São Paulo, SP, Brazil, 2020

**Items**	**Score distribution (%)**					**Mean ± SD** *
	**1**	**2**	**3**	**4**	**5**	
I have enough energy for my daily activities	1,8	3,8	26,9	34,7	32,9	3,9 ± 1,0
I do at least 2.5 hours of physical activity every week	34,1	21,2	19,8	9,2	15,6	2,5 ± 1,4
I feel rested when I awake	4,4	11,2	35,9	29,3	19,2	3,5 ± 1,1
I feel good about myself	1,4	3,8	24,6	36,1	34,1	4,0 ± 0,9
I am able to cope with the stresses in my life	1,6	4,8	29,7	36,5	27,5	3,8 ± 0,9
I have someone that I can talk to when I need help or support	3,4	4,0	18,0	18,2	56,3	4,2 ± 1,1
Religious or spiritual practices give meaning to my life	9,0	8,0	17,0	16,2	49,7	3,9 ± 1,3
I am comfortable with my sexuality	0,8	1,2	6,4	15,6	76,0	4,6 ± 0,7
My health is important to me	0,2	0,8	3,6	9,8	85,6	4,8 ± 0,6
I can make changes in my lifestyle to improve my health	0,6	2,4	16,4	25,5	55,1	4,3 ± 0,9
I control the types of food I eat	6,0	10,4	32,1	28,3	23,2	3,5 ± 1,1
I feel comfortable with my weight	12,8	13,0	24,6	22,8	26,7	3,3 ± 1,3
I heal easily	1,0	3,0	23,4	33,5	39,1	4,1 ± 0,9
I fall asleep without a problem	4,6	8,4	26,3	26,7	34,1	3,8 ± 1,1
I am hopeful about the future	1,2	2,0	12,6	22,6	61,5	4,4 ± 0,9
I feel in control of my life	1,6	4,0	24,0	38,1	32,3	4.0 ± 0,9
I like the way I look	1,6	3,6	20,8	36,3	37,7	4,0 ± 0,9
I feel good about the decisions I make	0,6	1,8	27,1	42,9	27,7	4,0 ± 0,9
I am satisfied with my problem-solving ability	0,8	4,0	25,7	36,7	32,9	4,0 ± 0,9
I seek immediate attention for changes in my health	4,0	10,8	23,4	26,3	35,5	3,8 ± 1,2
I am able to adjust to changes in my life	0,4	2,0	21,6	34,9	41,1	4,1 ± 0,9
I have an annual health examination	6,0	12,2	16,8	19,0	45,9	3,9 ± 1,3
I am able to follow recommendations from my health care provider	0,8	3,2	19,6	36,3	40,1	4,1 ± 0,9
I wear a seat belt	2,2	1,2	6,8	9,4	80,4	4,6 ± 0,8
I avoid the sun or use sunscreen	8,2	7,6	26,5	27,5	30,3	3,6 ± 1,2
I am in excellent health	1,6	4,6	22,4	37,3	34,1	4,0 ± 0,9
I am happy with my life	1,0	2,2	17,0	31,7	48,1	4,2 ± 0,9
I am able to hear clearly	0,2	0,8	10,6	22,4	65,9	4,5 ± 0,7
I can concentrate for a long period of time	1,6	6,2	24,8	37,1	30,3	3,9 ± 1,0
I am able to learn new information easily	0,4	2,6	17,8	34,1	45,1	4,2 ± 0,9
The choices I make about my life are consistent with my values	0,2	0,8	12,0	31,7	55,3	4,4 ± 0,7
I eat 5 to 6 servings of fruits and vegetables daily	15,6	26,1	32,5	20,0	5,8	2,7 ± 1,1
I drink 6 to 8 glasses of water a day	8,0	11,8	23,8	22,8	33,5	3,6 ± 1,3
I am satisfied with what I do for work	3,6	3,0	19,6	33,9	39,9	4,0 ± 1,0
I feel comfortable with the role I play in my family	2,4	2,6	18,6	33,1	43,3	4,1 ± 1,0
I am satisfied with my social life	1,8	3,8	22,0	30,5	41,9	4,1 ± 1,0
I feel comfortable expressing my emotions	2,8	5,0	24,8	29,7	37,7	3,9 ± 1,0
I feel I can easily communicate with others	0,8	5,0	16,8	30,5	46,9	4,2 ± 0,9
I have a usual routine that I perform to help me relax	4,6	11,0	30,3	30,3	23,8	3,6 ± 1,1
I consider myself to be healthy	3,2	9,6	25,9	32,1	29,3	3,7 ± 1,1
It is a burden to participate in caretaking activities	34,9	17,8	26,9	13,0	7,4	2,4 ± 1,3
I have difficulty urinating	76,2	9,8	5,2	4,2	4,6	1,5 ± 1,1
I have problems with bowel elimination	48,9	15,8	21,6	6,8	6,8	2,1 ± 1,3
I feel guilty when I drink alcohol, wine, or beer	55,9	14,6	13,2	5,2	11,0	2,0 ± 1,4
I use recreational drugs	87,2	5,0	4,8	1,8	1,2	1,2 ± 0,7
I smoke cigarettes	81,0	3,4	5,4	1,8	8,4	1,5 ± 1,2
I have difficulty with my vision	37,7	13,8	28,1	10,4	10,0	2,4 ± 1,3
My physical abilities limit my activities of daily living	51,7	14,2	20,0	8,8	5,2	2,0 ± 1,2
I have difficulty controlling my anger	29,9	24,2	28,5	10,6	6,8	2,4 ± 1,2
I feel physical symptoms with walking	49,3	14,2	22,0	9,0	5,4	2,1 ± 1,2
I worry a lot	8,0	8,0	27,1	23,2	33,7	3,7 ± 1,2
I feel at risk for physical harm	49,5	18,2	22,2	5,8	4,2	2,0 ± 1,2
I experience physical discomfort when I am under stress	27,9	15,8	29,1	14,6	12,6	2,7 ± 1,4
I feel stress	14,8	16,0	40,9	16,2	12,0	2,9 ± 1,2
I experience pain that interrupts my daily activities	44,5	21,6	20,4	7,6	5,8	2,1 ± 1,2
I have family problems that I find are difficult to handle	33,5	22,8	23,2	11,4	9,0	2,4 ± 1,3
I fear for my safety	8,0	6,8	22,2	18,2	44,7	3,8 ± 1,3

^*^ SD = Standard Deviation

### Confirmatory Factor Analysis and Internal Consistency

The factor loadings were significant and varied from weak to strong (0.16 to 0.75). All items had a variance estimation above zero, meaning that all of them contributed to estimate the model. The CFA confirmed a three-factor solution for the FHPAST-BR, reflecting all FHPs and yielding moderate fit ( Table 3). 

The internal consistency measured with Cronbach’s alpha was satisfactory for all three components, varying between 0.779 and 0.919. The Cronbach’s alpha values if each item is removed are shown in Table 3. The between-domain covariance showed a satisfactory correlation between Components 1 and 2 (0.536), Components 1 and 3 (0.345), and Components 2 and 3 (0.757). 

**Table 3 -:** Confirmatory Factor Analysis of the Functional Health Pattern Assessment Tool – Modified Brazilian Version (n = 499). São Paulo, SP, Brazil, 2020

**Items**		**Factor loadings**	**Cronbach’s Alpha if item removed**
**Component 1: Health Risk/Threat (Cronbach’s alpha: 0,795)**			
41	It is a burden to participate in caretaking activities	0,22	0,78
42	I have difficulty urinating	0,32	0,78
43	I have problems with bowel elimination	0,38	0,78
44	I feel guilty when I drink alcohol, wine, or beer	0,17	0,79
45	I use recreational drugs	0,20	0,79
46	I smoke cigarettes	0,25	0,78
47	I have difficulty with my vision	0,33	0,78
48	My physical abilities limit my activities of daily living	0,54	0,76
49	I have difficulty controlling my anger	0,54	0,77
50	I feel physical symptoms with walking	0,60	0,76
51	I worry a lot	0,57	0,77
52	I feel at risk for physical harm	0,53	0,77
53	I experience physical discomfort when I am under stress	0,65	0,76
54	I feel stress	0,61	0,77
55	I experience pain that interrupts my daily activities	0,62	0,77
56	I have family problems that I find are difficult to handle	0,52	0,77
57	I fear for my safety	0,16	0,79
**Component 2: General Well-Being & Self Confidence (Cronbach’s Alpha: 0,919)**			
1	I have enough energy for my daily activities	0,50	0,92
3	I feel rested when I awake	0,45	0,92
4	I feel good about myself	0,77	0,91
5	I am able to cope with the stresses in my life	0,63	0,91
6	I have someone that I can talk to when I need help or support	0,47	0,92
8	I am comfortable with my sexuality	0,37	0,92
12	I feel comfortable with my weight	0,40	0,92
13	I heal easily	0,58	0,91
14	I fall asleep without a problem	0,41	0,92
15	I am hopeful about the future	0,59	0,91
16	I feel in control of my life	0,66	0,91
17	I like the way I look	0,65	0,91
18	I feel good about the decisions I make	0,69	0,91
19	I am satisfied with my problem-solving ability	0,70	0,91
21	I am able to adjust to changes in my life	0,61	0,91
26	I am in excellent health	0,70	0,91
27	I am happy with my life	0,75	0,91
28	I am able to hear clearly	0,35	0,92
29	I can concentrate for a long period of time	0,51	0,92
30	I am able to learn new information easily	0,52	0,92
31	The choices I make about my life are consistent with my values	0,51	0,92
34	I am satisfied with what I do for work	0,45	0,92
35	I feel comfortable with the role I play in my family	0,59	0,91
36	I am satisfied with my social life	0,68	0,91
37	I feel comfortable expressing my emotions	0,58	0,91
38	I feel I can easily communicate with others	0,51	0,92
40	I consider myself to be healthy	0,69	0,91
Component 3: Health Promotion/Protection Activity (Cronbach’s Alpha: 0,779)			
2	I do at least 2.5h of physical activity every week	0,41	0,78
7	Religious or spiritual practices give meaning to my life	0,35	0,79
9	My health is important to me	0,50	0,77
10	I can make changes in my lifestyle to improve my health	0,53	0,77
11	I control the types of food I eat	0,62	0,76
20	I seek immediate attention for changes in my health	0,58	0,77
22	I have an annual health examination	0,46	0,77
23	I am able to follow recommendations from my health care provider	0,58	0,76
24	I wear a seat belt	0,28	0,79
25	I avoid the sun or use sunscreen	0,43	0,77
32	I eat 5 to 6 servings of fruits and vegetables daily	0,57	0,77
33	I drink 6 to 8 glasses of water a day	0,46	0,78
39	I have a usual routine that I perform to help me relax	0,60	0,77
CFI^*^ = 0,739			
TLI^†^ = 0,729			
RMSEA^‡^ = 0,057 (IC 90%^§^ = 0,055-0,059, p^||^ = 0.000)			
SRMR^¶^ = 0,062			

^*^ CFI = Confirmatory Factor Index;

^†^ TLI = Tucker Lewis Index;

^‡^ RMSEA = Root Mean Square Error of Approximation;

^§^ 90%CI = 90% Interval Confidence;

^||^ p = Significance Level;

^¶^ SRMR = Standardized Root Mean Square Residual

### Convergent validity

The correlation between the mean FHPAST scores and the question “Overall, how would you rate your current health?” was 0.48 (p < 0.001), whereas it was 0.44 (p < 0.001) between the mean FHPAST scores and the question “How satisfied are you with your current health?”.

## Discussion

We have shown that the FHPAST-BR is a valid and reliable tool to screen the FHPs in the Brazilian population. However, the CFA model only yielded moderate fit. FHAPAST was originally developed within the FHP framework and provides a holistic tool to screen “functional health and assess potential problems, risk and readiness for health” ^(^ ^8^ ^)^. It is a structured Nursing-driven assessment tool that fosters data collection relevant to Nursing. It provides a way of organizing these data to ease the decision-making process about the central elements of nursing practice, i.e., Nursing diagnoses, outcomes and interventions ^(^ ^11^ ^)^. 

Prior to assessing the internal structure, convergent validity and reliability, a revision of T1 was conducted, as well as content validation and evaluation of comprehensibility by individuals from the target population. The revision of T1 had two purposes. One of them was to update items according to more recent recommendations and to use words that were easier to read and understand. The second purpose was to conduct a lexical revision based on the double-route reading model. According to this model, reading is possible through two routes. One involves direct visual recognition of a word with rapid access to its meaning; this route requires memorizing letters and syllabi and a strong representation of the word in the orthographic lexicon. Reading through the phoneme route requires phonological decoding before gaining access to the meaning of the word ^(^ ^32^ ^-^ ^33^ ^)^. Although slower than the direct visual route, the phoneme route allows readers to read any word. Experimental models consistent with the double-route model show that longer words with more syllables and letters are more difficult to read and more likely to be misread ^(^ ^32^ ^)^. Noteworthy, the lexical revision conducted in this study allows for identifying words that are easier to read and more frequently used in a given country or culture. This may be used by other authors as a step in the process of revising previously translated/back-translated versions of measuring tools. 

For content validation, the number of experts and criteria we used to select the specialists were similar to other studies ^(^ ^34^ ^-^ ^35^ ^)^. Content validity ensured that FHPAST-BR contained an appropriate sample of items to assess the 11 FHPs, as all items were found to be relevant, and the revised items were considered precise. In the content validation of the Spanish version of the FHPAST, the authors found that the content validity index varied from 0.67 to 0.96 ^(^ ^10^ ^)^. Furthermore, the comprehensibility analysis by individuals from the target population was essential to guarantee that the items were understandable. Although no standardized methods exist to evaluate comprehensibility by individuals from the target population, this strategy has been used in cross-cultural validation studies ^(^ ^36^ ^-^ ^37^ ^)^. It is plausible to consider that the procedures used for content validation led to a low rate of missing data, with the exception of two items: “I am satisfied with what I do for work” (#34) and “I feel guilty when I drink alcohol, wine, or beer” (#44). Participants who did not have a formal job might not have answered item 34. Regarding item 44, it is likely that the stigma associated with alcohol consumption might have affected participants’ answers even though they filled out the FHPAST-BR tool on their own. 

Regarding the CFA, our sample differed from the one used by the authors of the previous Portuguese translation of the FHPAST ^(^ ^18^ ^)^, as ours included significant others, faculty and staff, in addition to patients and students. The CFA for the FHPAST-BR (57 items) confirmed that the three-factor solution was the best to measure the construct proposed in the Brazilian population. Nevertheless, six items showed low but significant loading factors (<0.30). Five of these items were loaded in Component 1: Health Risk/Threat, and one in Component 3: Health Promotion/Protection Activity. The low factor loadings mean that these items do not significantly contribute to explaining each factor. One possible explanation is that the items covered sensitive topics such as the use of seat belts (#24), caretaking activities (#41), drinking alcohol (#44), using recreational drugs (#45), smoking cigarettes (#46) and safety (#57), which might not be suitable to assess the FHP using a screening tool. Noteworthy, Cronbach’s alpha did not present significant changes if those items were removed. 

Further psychometric studies are necessary to explore how exclusion of these items will affect the internal structure of the FHPAST-BR. The Principal Component Analysis with Varimax rotation and Kaiser Normalization was used to test the internal structure of the English version of the FHPAST. The authors found that the three-component solution was the most parsimonious and interpretable. All 57 items had loading factors above 0.30 with few substantial side loadings ^(^ ^8^ ^)^. 

The FHP framework can be used to delineate one’s own overall health status ^(^ ^9^ ^)^. A cross-sectional study conducted during the COVID-19 pandemic found that changes in the FHP were correlated with anxiety levels and to self-perception of health. For instance, high anxiety scores were associated with changes in the health perception-health management pattern, which was explained by the adoption of protective behaviors such as hand washing ^(^ ^38^ ^)^. In the current study, convergent validity revealed a positive, moderate and significant relationship between the FHPAST-BR score and self-perception and satisfaction with current health status, suggesting that the FHPAST-BR measures the construct that it is intended to be measured. In the Spanish validation study, the FHPAST was positively, moderately and significantly correlated with two quality-of-life indices ^(^ ^10^ ^)^. 

In our study, all three components had satisfactory internal consistency. Component 3 had the lowest Cronbach’s alpha (0.779), whereas Component 2 had the highest alpha (0.919). In the validation study of the original tool, the authors also found that each component had satisfactory internal consistency ^(^ ^8^ ^)^. In the Spanish validation, the authors only obtained the global Cronbach’s alpha, which was also acceptable ^(^ ^10^ ^)^. In addition, they found that the half-half correlation supported evidence of internal consistency. Altogether, these findings show the robustness of the FHPAST across cultures. 

This study has limitations that need to be considered when interpreting the data. First, although the level of missing data was low for each item, in total, 30% of the initial sample (n = 218/717) did not answer at least one item. This might indicate that the tool is too long or that the instrument lacks face validity, which was not assessed before data collection. Secondly, no gold standard to assess functional health patterns is available in the literature. In order to evaluate convergent validity, we used two questions concerning self-perception of and satisfaction with health status. We used those two questions instead of a standardized tool to avoid response fatigue and eliminate the possible time constraints that would have discouraged participation in this study. Thirdly, the measuring scale was modified from a 4-point to a 5-point scale and the participants were not provided with a definition of each answer category. Although for most questions the participants selected options to the right or left extremes of the scale, it is possible that the midpoint response bias and the absence of an operational definition for the scale categories might have affected fit of the model. Lastly, we acknowledge that, despite our efforts to recruit a diverse sample, most of the participants were women and reported having High School education or higher levels. These factors might exert impacts on the external validity of our findings. Future studies should test the FHPAST-BR by removing the items with low factor loadings and adding a definition for each answer category in a more diverse sample. Although continuing refinement, testing and validation of the FHPAST-BR is necessary, this study brings about important contributions for Nursing practice and for advancing Nursing knowledge. An FHP screening assessment tool is innovative in the Brazilian Nursing practice. The FHPAST-BR has the potential to guide clinical decision-making, helping nurses in different clinical settings to identify patient problems accurately and in a timely manner.

## Conclusion

The CFA confirmed the three-factor solution, as found in the English version of the FHPAST. Six items had low (<0.30) loading factors, and the model only yielded moderate fit. However, the three components of the FHPAST-BR showed evidence of convergent validity and satisfactory internal consistency. Hence, the FHPAST-BR has validity and reliability evidence to assess the FHPs in the Brazilian population, although caution is recommended when interpreting the results due to the moderate fit of the model. The FHPAST-BR may be used in clinical practice and research. Use of the tool is free of charge but permission from the first author is required.

## References

[1] American Nurses Association (2021). Nursing: scope and standards of practice.

[2] Narayan M. C. (2022). What constitutes patient-centered care in home care? A descriptive study of home health nurses’ attitudes, knowledge, and skills. Home Health Now.

[3] Institute of Medicine of the National Academies (2010). The future of nursing: leading the change, advancing health.

[4] Kennerly S. M., Sharkey P. D., Horn S. D., Alderden J., Yap T. L. (2022). Nursing assessment of pressure injury risk with the Braden scale validated against sensor-based measurement of movement. Healthcare.

[5] Wang L., McArthur A., Lu Z., Yang Y., Lu H., Chen F. (2020). Prechemotherapy nursing assessment among adult cancer patients in a university cancer center in Shanghai, China: a best practice implementation project. JBI Evid Implement.

[6] Butcher R. C. G. S., Jones D. A. (2021). An integrative review of comprehensive nursing assessment tools developed based on Gordon’s Eleven Functional Health Patterns. Int J Nurs Knowl.

[7] DeSanto-Madeya S. (2007). Using case studies based on a nursing conceptual model to teach medical-surgical nursing. Nurs Sci Q.

[8] Jones D., Duffy M. E., Flanagan J., Foster F. (2012). Psychometric evaluation of the Functional Health Pattern Assessment Screening Tool (FHPAST). Int J Nurs Knowl.

[9] Gordon M. (1994). Nursing diagnosis: process and application.

[10] Sánchez I. S., Brito P. R. B., Muñoz M. N., Gómez J. A. R. (2021). Spanish validation of the Functional Health Pattern Assessment Screening Tool (FHPAST) in primary health care. Rev Cuidar [Internet].

[11] Jones D. A., Herdman T. H., Butcher R. C. G. S., Herdman T. H., Kamitusuru S., Lopes C. T. (2021). NANDA International nursing diagnoses: definitions and classifications 2021-2023.

[12] Gasperini B., Pelusi G., Frascati A., Sarti D., Dolcini F., Espinosa E. (2021). Predictors of adverse outcomes using a multidimensional nursing assessment in an Italian community hospital. PLoS One.

[13] Cosolo L., Leahey A., Elmi S., Homeward T. (2023). Development of a nurse-initiated proactive telephone nursing assessment guideline for new cancer patients. Can Oncol Nurs J.

[14] Carneiro C. S., Lopes J. L., Herdman T. H., Lopes C. T., Bachion M. M., Barros A. L. B. L. (2014). Construction and validation of a data collection tool for the clinical assessment of human responses of outpatients with chronic cardiovascular diseases. Int J Nurs Knowl.

[15] Zega M., D’Agostino F., Bowles K. H., De Marinis M. G., Rocco G., Vellone E. (2014). Development and validation of a computerized assessment form to support nursing diagnosis. Int J Nurs Knowl.

[16] Paans W., Müller-Staub M. (2015). Patients’ care needs: documentation analysis in general hospitals. Int J Nurs Knowl.

[17] Rodrigues A. B., Cunha G. H., Aquino C. B. Q., Rocha SR, Mendes CRS, Firmeza M. A. (2018). Head and neck cancer: validation of a data collection instrument. Rev Bras Enferm.

[18] Barros A. L. B. L., Michel J. L. M., Nobrega M. M. L. (2003). Translation, utilization, and psychometric properties of the Functional Health Pattern Assessment Screening Tool with patients in Brazil. Int J Nurs Terminol Classif.

[19] Boateng G. O., Neilands T. B., Frongillo E. A., Melgar-Quiñonez H. R., Young S. L. (2018). Best practices for developing and validating scales for health, social, and behavioral research: a primer. Front Public Health.

[20] Brislin R. N. (1970). Back-translation for cross-cultural research. J Cross Cult Psychol.

[21] Simão A. F., Precoma D. B., Andrade J. P., Correa H., Saraiva J. F. K., Oliveira G. M. M. (2003). I Diretriz Brasileira de Prevenção Cardiovascular. Arq Bras Cardiol.

[22] Milin P., Divjak D., Baayen R. H. (2017). A learning perspective on individual differences in skilled reading: exploring and exploiting orthographic and semantic discrimination cues. J Exp Psychol Learn Mem Cogn.

[23] Corpus Brasileiro [Homepage] http://corpusbrasileiro.pucsp.br/cb/Inicial.html.

[24] Cortela C. C., Milistetd M., Both J., Gonçalves G. H. T., Balbinotti C. A. A. (2020). Validação da escala de contextos de aprendizagem para treinadores esportivos - versão tênis. Rev Bras Ciênc Esporte.

[25] Vergara Escobar OJ, Carrillo González GM (2023). Self-management program in adults with colorectal cancer: a pilot study. Aquichan.

[26] Ferreira N. C., Moorhead S., Butcher R. C. G. S. (2021). The nurse–patient outcome content validation method. Int J Nurs Terminol Knowl.

[27] Pasquali L., Pasquali L. (2010). Instrumentação psicológica: fundamentos e práticas.

[28] Engler K., Vicente S., Mate K. K. V., Lessard D., Ahmed S., Lebouché B. (2022). Content validation of a new measure of patient-reported barriers to antiretroviral therapy adherence, the I-Score: results from a Delphi study. J Patient Rep Outcomes.

[29] DeVelis R. F. (2021). Scale development: theory and applications.

[30] Ondé D., Alvarado J. M. (2020). Reconsidering the conditions for conducting confirmatory factor analysis. Span J Psychol.

[31] Tavakol M., Dennick R. (2011). Making sense of Cronbach’s alpha. Int J Med Educ.

[32] Capovilla F. C., Capovilla A. G. S., Macedo E. C., Pasquali L. (2010). Instrumentação psicológica: fundamentos e práticas.

[33] Cardoso H. S. P., Freitas P. M. (2019). Aplicação do modelo da dupla rota no diagnóstico da dislexia: revisão sistemática. Rev Psicopedag [Internet].

[34] Afonso B. Q., Ferreira N. D. C., Butcher R. C. G. S. (2020). Content validation of the symptom control outcome for heart failure patients in palliative care. Rev Gaucha Enferm.

[35] Afonso B. Q., Ferreira N. C., Butcher R. C. G. S. (2020). Conceptual and operational definitions for the indicators of the nursing outcome classification: symptom control in patients with heart failure in palliative care. Enferm Clin (Engl Ed).

[36] Conti M. A., Latorre M. R., Hearst N., Segurado A. (2009). Cross-cultural adaptation, validation and reliability of the Body Area Scale for Brazilian adolescents. Cad Saúde Pública.

[37] Kourakou A., Tigani X., Bacopoulou F., Vlachakis D., Papakonstantinou E., Simidala S. (2021). The Rosenberg Self-Esteem Scale: translation and validation in the Greek language in adolescents. Adv Exp Med Biol.

[38] Alan S., Gokyildiz Surucu S., Avcibay Vurgec B., Cevik A. (2021). An investigation of individuals’ health anxiety during the COVID-19 pandemic within the framework of the functional health patterns. Perspect Psychiatr Care.

